# Dual inhibition of thioredoxin reductase and proteasome is required for auranofin-induced paraptosis in breast cancer cells

**DOI:** 10.1038/s41419-023-05586-6

**Published:** 2023-01-19

**Authors:** Min Ji Seo, In Young Kim, Dong Min Lee, Yeon Jung Park, Mi-Young Cho, Hyo Joon Jin, Kyeong Sook Choi

**Affiliations:** 1 Department of Biochemistry and Molecular Biology, Ajou University School of Medicine, Suwon, 16499 Korea; 2 Department of Biomedical Sciences, Ajou University Graduate School of Medicine, Suwon, 16499 Korea; 3 Nano-safety Team, Safety Measurement Institute, Korea Research Institute of Standards and Science (KRISS), Daejeon, 34113 Korea; 4 Ajou University School of Medicine, Suwon, 16499 Korea

**Keywords:** Cell death, Stress signalling

## Abstract

Auranofin (AF), a gold (I)-containing phosphine compound, is being investigated for oncological application as a repurposed drug. We show here that 4~5 µM AF induces paraptosis, a non-apoptotic cell death mode characterized by dilation of the endoplasmic reticulum (ER) and mitochondria, in breast cancer cells. Although the covalent inhibition of thioredoxin reductase (TrxR), an enzyme that critically controls intracellular redox homeostasis, is considered the primary mechanism of AF’s anticancer activity, knockdown of TrxR1 did not induce paraptosis. Instead, both TrxR1 knockdown plus the proteasome inhibitor (PI), bortezomib (Bz), and 2 μM AF plus Bz induced paraptosis, thereby mimicking the effect of 5 μM AF. These results suggest that the paraptosis induced by 5 μM AF requires the inhibition of both TrxR1 and proteasome. We found that TrxR1 knockdown/Bz or subtoxic doses of AF and Bz induced paraptosis selectively in breast cancer cells, sparing non-transformed MCF10A cells, whereas 4~5 μM AF killed both cancer and MCF10A cells. GSH depletion was found to be more critical than ROS generation for the paraptosis induced by dual TrxR1/proteasome inhibition. In this process, the ATF4/CHAC1 (glutathione-specific gamma-glutamylcyclotransferase 1) axis leads to GSH degradation, contributing to proteotoxic stress possibly due to the accumulation of misfolded thiol-containing proteins. These results suggest that the paraptosis-inducing strategy of AF plus a PI may provide an effective therapeutic strategy against pro-apoptotic therapy-resistant cancers and reduce the potential side effects associated with high-dose AF.

## Introduction

Auranofin (AF), an FDA-approved antirheumatic drug [1], has been targeted for drug repurposing in anticancer therapy with encouraging results [2]. Reports indicate that thioredoxin reductase (TrxR) is a primary target in the anticancer effect of AF [3–5]. TrxR (TrxR1 in the cytosol and TrxR2 in mitochondria) is a key member of the thioredoxin (Trx) system, in which TrxR, Trx, and NADPH contribute to redox regulation by mediating the reduction of disulfide bonds [3, 4, 6]. AF may also elicit cytotoxicity in cancer cells by disrupting protein homeostasis (proteostasis) via proteasomal inhibition, in particular, by targeting the 19 S proteasome-associated deubiquitinases (DUBs), UCHL5, and USP14 [7–9]. We show here for the first time that 4~5 μM AF induces paraptosis in breast cancer cells by dually targeting TrxR1 and proteasome.

Paraptosis is a non-apoptotic cell death mode accompanied by dilation of the ER and/or mitochondria [10, 11]. Although apoptosis has been considered a major mechanism of chemotherapy-induced cell death, many tumors often develop resistance to most apoptosis-based cancer therapies [12, 13]. Therefore, targeting non-apoptotic forms of cell death, such as paraptosis, may have therapeutic benefits in apoptosis-defective cancer cells. Although the molecular basis of paraptosis remains to be clarified, it has been causatively linked to disruption of proteostasis, such as through inhibition of thiol proteostasis [11, 14–17] or proteasome [18–21]. We found that mono-inhibition of TrxR1 or proteasome does not induce a notable anticancer activity. Instead, inhibition of both TrxR1 and proteasome demonstrates an effective anticancer activity by inducing paraptosis, suggesting that AF-induced paraptosis results from the dual inhibition of TrxR1 and proteasome.

Successful cancer therapy must selectively kill cancer cells while sparing normal cells. AF at 4~5 μM was found to kill both breast cancer and non-malignant breast epithelial MCF10A cells, suggesting the potential for side effects in clinical use. In contrast, a combined regimen of 2 μM AF plus and the proteasome inhibitor (PI), bortezomib (Bz), or TrxR1 knockdown plus Bz induced paraptosis selectively in breast cancer cells, sparing MCF10A cells. Mechanistically, dual TrxR1/proteasome inhibition upregulates the ATF4/CHAC1 axis to degrade GSH, thereby contributing to at least a portion of the paraptosis by aggravating proteotoxic stress.

## Results

### Auranofin at 4-5 μM triggers paraptosis in breast cancer cells

**Fig. 1:**
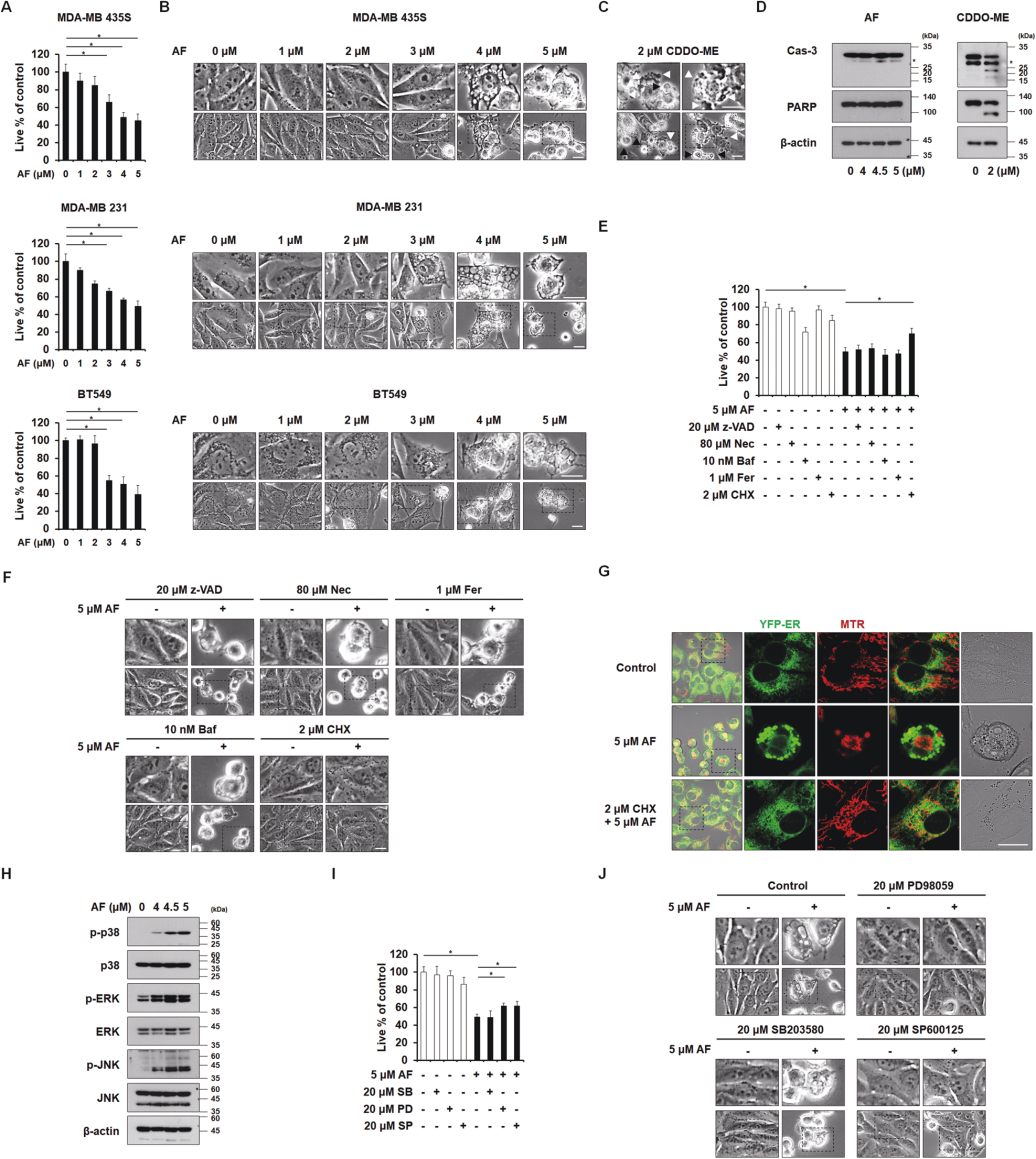
Auranofin induces paraptosis in several breast cancer cell lines. **A**–**D** Cells were treated with the indicated concentrations of AF or CDDO-ME for 24 h. **E**, **F** MDA-MB 435 S cells pretreated with the indicated doses of z-VAD-fmk (z-VAD), necrostatin-1 (Nec), ferrostatin-1 (Fer), 3-methyladenine (3-MA), bafilomycin A1 (Baf), or cycloheximide (CHX) were further treated with 5 μM AF for 24 h. **A**, **E** Cellular viability was assessed using IncuCyte, as described in the Materials and Methods. The percentage of live cells was normalized to that of untreated cells (100%). Data represent the means ± SD. (*n* = 9). One way-ANOVA and Bonferroni’s post hoc test. ^*^p < 0.01. **B**, **C**, **F** Cellular morphologies were observed by phase-contrast microscopy. White and black arrow heads denote blebbing and apoptotic body, respectively (**C**). Bars, 20 μm. **D** Western blotting of caspase-3 and PARP was performed using β-actin as a loading control. **G** YFP-ER cells treated with 5 μM AF and/or 2 μM CHX for 24 h were stained with MitoTracker-Red (MTR). Cells were observed by confocal microscopy. Bars, 20 μm. **H** MDA-MB 435 S cells were treated with the indicated concentrations of AF for 12 h, and Western blotting of the indicated proteins was performed using β-actin as a loading control. **I**, **J** MDA-MB 435 S cells pretreated with the indicated inhibitors were further treated with 5 μM AF for 24 h. **I** Cellular viability was assessed using IncuCyte. The percentage of live cells was normalized to that of untreated cells (100%). Data represent the means ± SD. (*n* = 9). One way-ANOVA and Bonferroni’s post hoc test. ^*^p < 0.05. **J** Cellular morphologies were observed by phase-contrast microscopy. Bars, 20 μm.

To investigate the effect of AF on breast cancer cells, we treated several breast cancer cell lines with various concentrations of AF. We found that AF treatment dose-dependently induced cell death accompanied by extensive vacuolation in MDA-MB 435 S, MDA-MB 231, and BT549 cells, exhibiting IC_50_ values of 4.71 μM, 4.85 μM, and 4.17 μM, respectively (Fig. 1A, B). Since we previously reported that 2-cyano-3,12-dioxooleana-1,9(11)-dien-C28-methyl ester (CDDO-Me) induces apoptosis accompanied by ER-derived vacuolation in breast cancer cells [22], we examined the possible involvement of apoptosis in the anticancer effect of AF. Interestingly, the morphological features of apoptosis induced by CDDO-ME [23], including cellular shrinkage, blebbing, and apoptotic body formation, were not observed in AF-treated cancer cells (Fig. 1B, C). In addition, unlike CDDO-ME, the application of AF (up to 5 μM) to MDA-MB 435 S cells did not lead to the cleavage of caspase-3 or its substrate, PARP (Fig. 1D). Pretreatment with the pan-caspase inhibitor, z-VAD-fmk (z-VAD), did not significantly affect AF-induced cell death or vacuolation in the tested cancer cells (Fig. 1E, F, and Supplementary Fig. 1). The morphological features of necroptosis, including cytoplasmic swelling and cell rupture, were not observed in cells treated with 4~5 μM AF (Fig. 1B), and pretreatment with necrostatin-1 (Nec; a necroptosis inhibitor) did not significantly affect AF-induced cell death or vacuolation (Fig. 1E, F, and Supplementary Fig. 1). Moreover, pretreatment with ferrostatin-1 (Fer; a ferroptosis inhibitor) or bafilomycin A1 (Baf; a late-phase autophagy inhibitor) failed to inhibit AF-induced cell death and vacuolation in these cells (Fig. 1E, F, and Supplementary Fig. 1). Our results suggest that the antitumor effect of 4~5 μM AF on the tested cells may depend more heavily on a cell death mode other than apoptosis, necroptosis, ferroptosis, or autophagy-dependent cell death. Since paraptotic cell death is accompanied by vacuolation derived from dilation of the ER and/or mitochondria [10, 11], we investigated whether AF induces paraptosis. Observation of the ER and mitochondria employing YFP-ER cells (stably expressing fluorescence in the ER lumen) [18] and MitoTracker-Red (MTR; a fluorescent stain for mitochondria) revealed that untreated cells had the expected reticular-shaped ER and filamentous mitochondria (Fig. 1G), but AF-treated cells exhibited vacuoles derived from dilated ER and mitochondria, a morphological feature of paraptosis. Since paraptosis requires *de novo* protein synthesis [10], we next tested the effect of the protein synthesis blocker, cycloheximide (CHX), on AF-induced cell death. We found that CHX effectively blocked AF-induced cell death and vacuolation in MDA-MB 435 S, MDA-MB 231, and BT549 cells (Fig. 1E, F, and Supplementary Fig. 1). CHX also effectively blocked AF-induced vacuolation derived from the ER and mitochondria (Fig. 1G). MAP kinases are associated with paraptosis induced by curcumin [18], celastrol [24], and gambogic acid [15]. We also found that AF activated p38, ERKs, and JNKs in MDA-MB 435 S cells (Fig. 1H). An experiment designed to test the functional significance of MAP kinases in this process showed that pretreatment with PD980596 (an inhibitor of MEK/ERK pathway) or SP600125 (a JNK inhibitor) partially but significantly inhibited AF-induced cell death in MDA-MB 435 S cells and effectively attenuated vacuolation. In contrast, these parameters were not affected by treatment with SB203580 (a p38 MAPK inhibitor) (Fig. 1I, J). These results suggest that activation of ERKs and JNKs may be critically involved in AF-induced paraptosis. Taken together, paraptosis may crucially contribute to the killing effect of 5 μM AF in these cancer cells.

### AF-induced paraptosis requires inhibition of both TrxR1 and proteasome

**Fig. 2:**
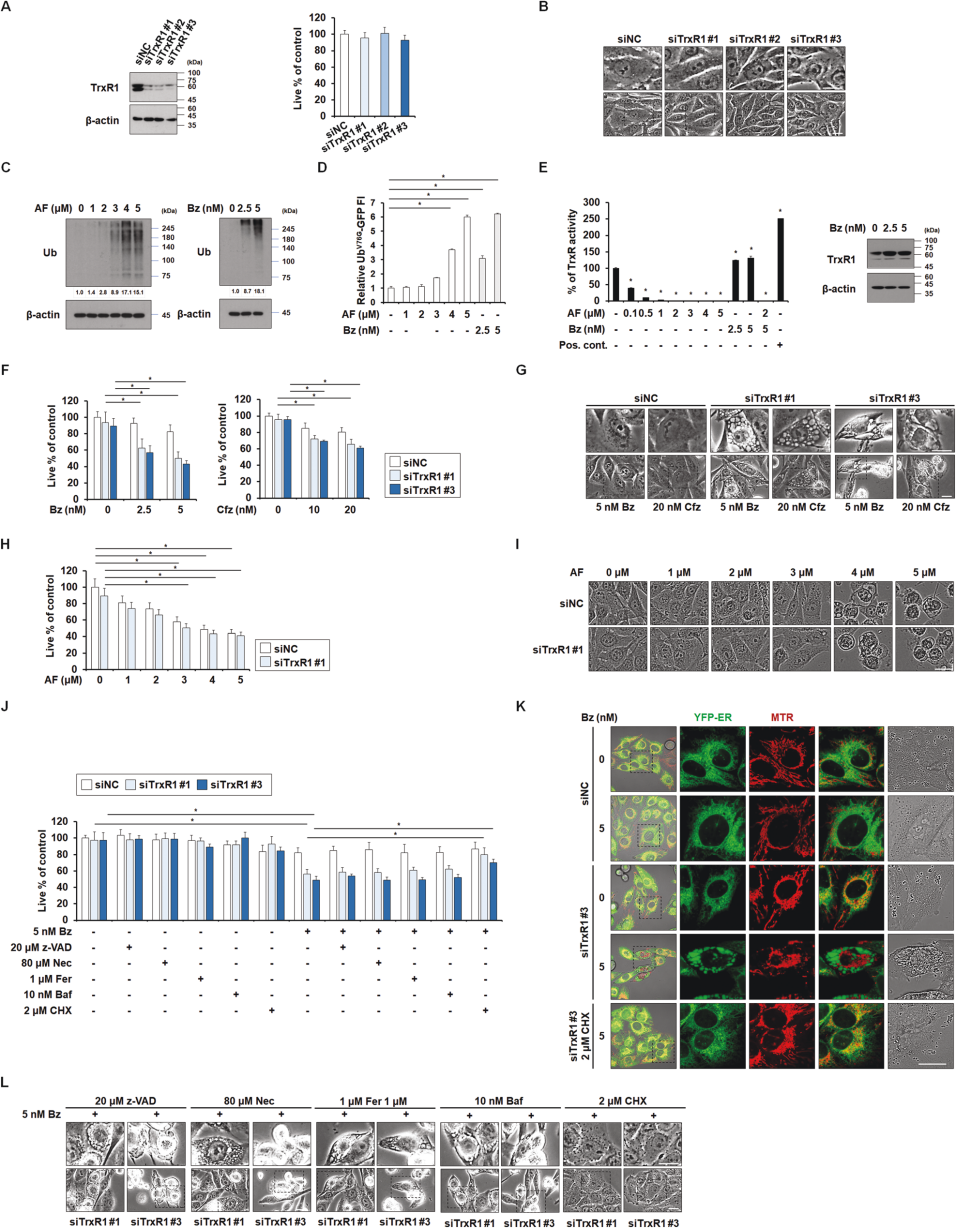
TrxR1/proteasome inhibition is required for AF-induced paraptosis. **A**, **B** MDA-MB 435 S cells were transfected with the negative control siRNA (siNC) or three different siRNAs against TrxR1 (siTrxR1) for 24 h. **A** Knockdown of TrxR1 was confirmed by Western blotting with β-actin used as a loading control (*left*). Cellular viability in transfected cells was assessed using IncuCyte, as described in the Materials and Methods. The percentage of live cells was normalized to that of untreated cells (100%). Data represent the means ± SD. (*n* = 9). One way-ANOVA and Bonferroni’s post hoc test (*right*). **B** Morphologies of the transfected cells were observed by phase-contrast microscopy. Bars, 20 μm. **C** MDA-MB 435 S cells were treated with the indicated concentrations of AF (*left*) or Bz (*right*) for 12 h. Western blotting of the ubiquitinated proteins was performed using β-actin as a loading control. **D** MDA-MB 435 S cells transfected with Ub^G76V^-GFP were treated with the indicated concentrations of AF or Bz for 12 h. The fluorescence intensity was assessed, as described in the Materials and Methods. **E** TrxR1 activity assay was performed in MDA-MB 435 S cells treated with AF and/or Bz at the indicated concentrations for 20 min as described in Materials and Methods (*left*). Rat liver TrxR was used as a positive control (Pos. cont.). The percentage of TrxR activity was normalized to that of untreated cells (100%). Data represent the means ± SEM. (*n* = 3). One way-ANOVA and Dunn’s test. ^*^p < 0.001. Changes in TrxR1 protein levels following treatment with 2.5 or 5 nM Bz were examined by Western blotting usig β-actin as a loading control (*right)*. **F**, **G** MDA-MB 435 S cells were transfected with siNC or siTrxR1 for 24 h and further treated with the indicated doses of Bz or Cfz. **H**, **I** MDAS-MB 435 S cells transfected with siNC or siTrxR1 were further treated with the indicated concentrations of AF for 24 h. **J**, **K** MDA-MB 435 S cells were transfected with siNC or siTrxR1 for 24 h, pretreated with the indicated doses of z-VAD, Nec, Fer, 3-MA, Baf, or CHX, and further treated with 5 nM Bz for 24 h. **F**, **H**, **J** Cellular viability was assessed using IncuCyte, as described in the Materials and Methods. The percentage of live cells was normalized to that of untreated cells (100%). Data represent the means ± SD. (*n* = 9). One way-ANOVA and Bonferroni’s post hoc test. ^*^p < 0.001 (**F**), ^*^p < 0.05 (**H**), ^*^p < 0.05 (**J**). Cellular morphologies were observed by phase-contrast microscopy (**G**, **L**) or employing IncuCyte (**I**). Bars, 20 μm. **K** YFP-ER cells transfected with siNC or siTrxR1 were further treated with 5 nM Bz and/or 2 μM CHX for 24 h and then stained with MTR. Cells were observed by confocal microscopy. Bars, 20 μm.

Next, we investigated whether AF induces paraptosis via inhibition of TrxR1. TrxR1 knockdown using three independent siRNAs did not alter the viability or morphology of MDA-MB 435 S cells (Fig. 2A, B), suggesting that AF-mediated TrxR1 inhibition would not be enough to trigger paraptosis. Since AF was also shown to inhibit proteasome [7–9], we examined the involvement of proteasome inhibition in AF-induced paraptosis [7]. An increase in ubiquitylated proteins is a hallmark of proteasome inhibition [25], and 4~5 μM AF increased the ubiquitylated protein levels, with an effect similar to that of the PI (Bz, 5 nM) (Fig. 2C). We further measured proteasome activity using the Ub^G76V^-GFP reporter, which contains a single uncleavable N-terminally linked ubiquitin that is attached to GFP and acts as a substrate for polyubiquitination and proteasome-mediated proteolysis [26, 27]. We found that 5 μM AF inhibits proteasome to the same extent as 5 nM Bz (Fig. 2D). TrxR activity assays revealed that AF effectively inhibited TrxR1 activity, with almost complete inhibition seen in groups treated with 2~5 μM AF (Fig. 2E). Treatment with 2.5 or 5 nM Bz slightly increased TrxR activity, possibly due to an increase in TrxR1 protein levels. However, TrxR activity was effectively suppressed in cells treated with 5 nM Bz plus 2 μM AF. These results suggest that AF at the doses that induce paraptosis (4~5 μM) inhibits both TrxR and proteasome, whereas its cytostatic doses (1~2 μM) mainly inhibit TrxR. Previously, we reported that proteasome inhibition is necessary but not sufficient to induce paraptosis, suggesting the requirement of other additional signals [19, 20, 28, 29]. Therefore, we examined whether dual TrxR1/proteasome inhibition could mimic 4~5 μM AF’s paraptosis-inducing activity. We found that while treatment with a PI alone (up to 5 nM Bz or 20 nM carfilzomib (Cfz)) did not notably induce cell death in MDA-MB 435 S cells, TrxR1 knockdown plus Bz or Cfz significantly reduced cell viability and induced vacuolation (Fig. 2F, G). Next, we examined whether TrxR1 knockdown affects the cellular responses to various doses of AF. Compared to the transfection with the siRNA Negative Control (siNC), TrxR1 knockdown slightly reduced the cell viability in cells treated with 1~5 μM AF (Fig. 2H). However, neither the morphology of cells treated with 1~3 μM AF nor the vacuolation induced by 4 or 5 μM AF was affected by TrxR1 knockdown (Fig. 2I). These results suggest again that AF-induced paraptosis cannot be explained by TrxR1 inhibition alone, and that additional proteasome inhibition is required. CHX, but not inhibitors of the other death modes, effectively inhibited the vacuolation-associated cell death induced by TrxR1 knockdown plus Bz (TrxR1 knockdown/Bz) (Fig. 2J, L). Furthermore, TrxR1 knockdown/Bz, but not either mono-treatment, induced dilation of the ER and mitochondria, and CHX pretreatment effectively inhibited these effects (Fig. 2K). Thus, the co-treatment yielded an effect similar to that obtained with 5 μM AF (Fig. 1E-G). These results suggest that AF at 4~5 μM induces paraptosis by inhibiting both TrxR1 and proteasome.

### AF at 4~5 μM kills breast cancer and non-transformed cells but lower-dose AF plus Bz spares non-transformed cells

**Fig. 3:**
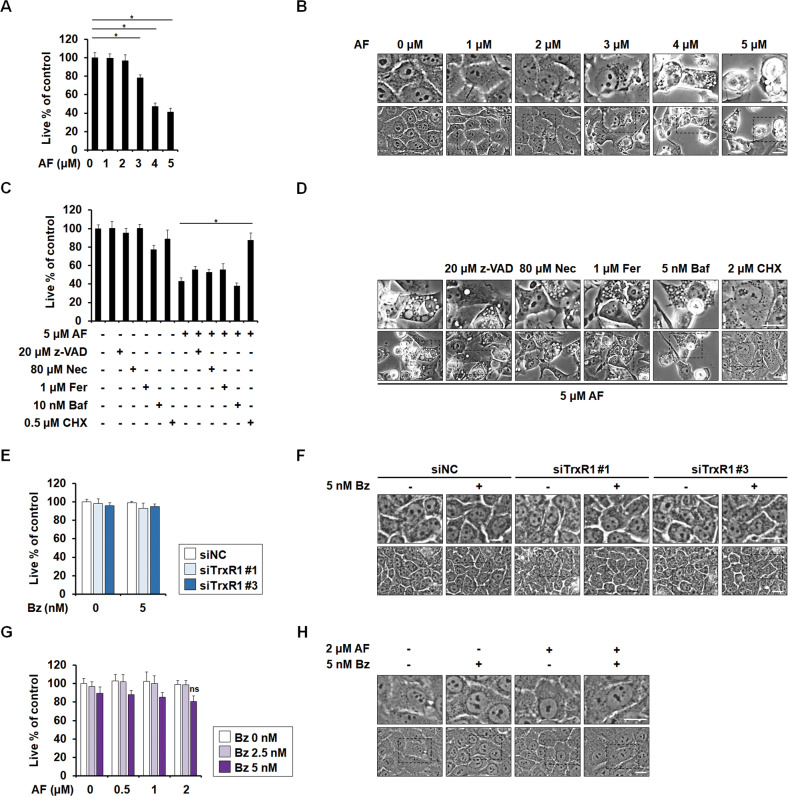
Non-transformed cells are killed by 4~5 μM AF, but spared by TrxR1 knockdown plus Bz or 2 μM AF plus Bz. **A**, **B** MCF10A cells were treated with the indicated concentrations of AF for 24 h. **C**, **D** MCF10A cells pretreated with the indicated doses of z-VAD, Nec, Fer, 3-MA, Baf, or CHX were further treated with AF for 24 h. **E**, **F** MCF10A cells transfected with siNC or siTrxR1 for 24 h were further treated with the indicated doses of Bz for 24 h. **G**, **H** MCF10A cells were treated with the indicated concentrations of AF and/or Bz for 24 h. **A**, **C**, **E**, **G** Cellular viability was assessed using IncuCyte, as described in the Materials and Methods. The percentage of live cells was normalized to that of untreated cells (100%). Data represent the means ± SD. (*n* = 9). One way-ANOVA and Bonferroni’s post hoc test. * p < 0.001; ns (non-significant). **B, D, F, H** Cellular morphologies were observed by phase-contrast microscopy. Bars, 20 μm.

Next, we tested whether AF preferentially kills cancer cells over normal cells. We found that 4~5 μM AF significantly reduced viability and induced vacuolation in non-transformed breast epithelial MCF-10A cells (Fig. 3A, B), and thus is not cancer-selective. Treatment of MCF10A cells with 4 μM AF demonstrated a level of cytotoxicity (about 55%) (Fig. 3A) similar to that obtained with 5 μM AF in MDA-MB 435 S cells (Fig. 1A). The cell death of MCF10A cells treated with 4 μM AF was significantly inhibited by CHX and weakly, but not significantly, attenuated by z-VAD, Nec, or Fer (Fig. 3C). AF-induced vacuolation was inhibited only by CHX (Fig. 3D). These results suggest that 4~5 μM AF may induce paraptosis as part of a mixed type of cell death in MCF10A cells, whereas it kills MDA-MB 435 S cells mainly by inducing paraptosis. Interestingly, the viability and morphology of MCF10A cells were not affected by TrxR1 knockdown or 2 μM AF, in the presence or absence of 5 nM Bz (Fig. 3E–H). These results suggest that combined sub-lethal doses of AF and PI may yield anticancer effects without the cytotoxicity toward normal cells, in contrast to high-dose AF.

### Subtoxic doses of AF and PI synergistically induce paraptosis in breast cancer cells

**Fig. 4:**
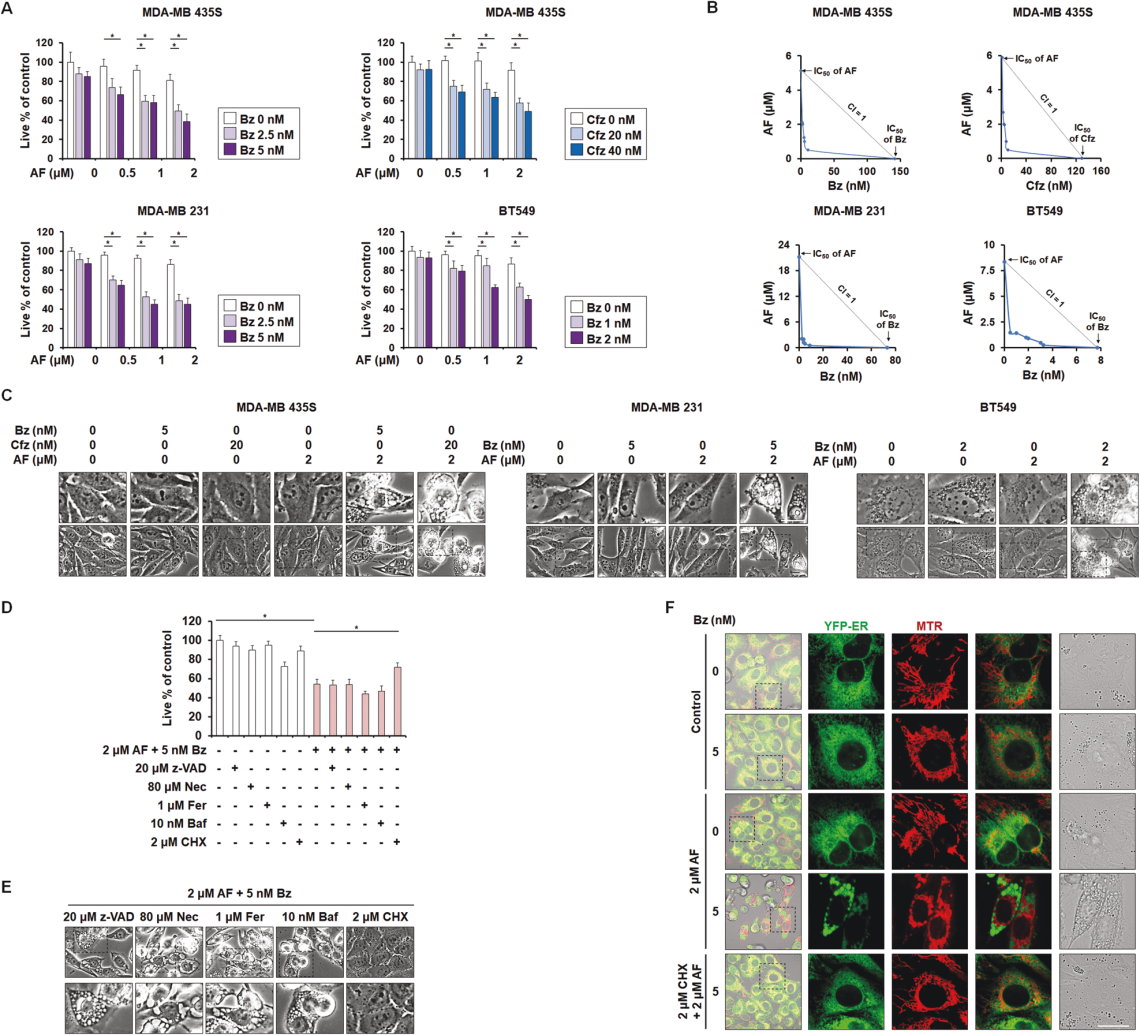
Combining low doses of AF and PI induces paraptosis in breast cancer cells. **A**–**C** Cells were treated with the indicated concentrations of AF and/or PIs for 24 h. **D**, **E** MDA-MB 435 S cells pretreated with the indicated dose of z-VAD, Nec, Fer, 3-MA, Baf, or CHX were further treated with 2 μM AF plus 5 nM Bz for 24 h. **A**, **D** Cellular viability was assessed using IncuCyte, as described in the Materials and Methods. The percentage of live cells was normalized to that of untreated cells (100%). Data represent the means ± SD. (*n* = 9). One way-ANOVA and Bonferroni’s post hoc test. ^*^p < 0.05. B. **B** Isoboles for the combinations of AF and Bz or AF and Cfz that proved iso-effective (IC_50_) for inhibiting cell viability in the tested cancer cells. **C**, **E** Cellular morphologies were observed by phase-contrast microscopy. Bars, 20 μm. **F** YFP-ER cells treated with 2 μM AF plus 5 nM Bz and/or 2 μM CHX for 24 h were stained with MTR. Cells were observed by confocal microscopy. Bars, 20 μm.

Next, we investigated whether combining low doses of AF and PI induced paraptosis in MDA-MB 435 S cells, as seen for TrxR1 knockdown plus PI. Indeed, sub-lethal doses of AF significantly reduced cell viability when combined with Bz or Cfz, demonstrating that AF and PI have a synergistic effect (Fig. 4A, B). In addition, 2 μM AF dramatically induced vacuolation when combined with 5 nM Bz or 20 nM Cfz (Fig. 4C). Similar results were observed in MDA-MB 231 and BT-549 cells treated with AF plus Bz, although a lower dose of Bz was required to sensitize AF-mediated anticancer effects in BT-549 cells (Fig. 4A–C). These results suggest that the anticancer effect of low-dose AF plus PI may not be restricted to a particular cancer cell line. We also found that 2 μM AF plus 5 nM Bz induced paraptosis in MDA-MB 435 S cells, similar to the effect of TrxR1 knockdown plus 5 nM Bz (Fig. 4D–F). In these experiments, 2 μM AF mimicked the paraptosis-sensitizing effect of TrxR1 knockdown in Bz-treated cells (Fig. 2F–I), indicating that 2 μM AF may inhibit TrxR1 as a major target. Collectively, these results suggest that a combination of low-dose AF and PI preferentially kills breast cancer cells by inducing paraptosis, providing a cancer-selective therapeutic strategy that should have fewer side effects than high-dose AF.

### GSH depletion is critical for the paraptosis induced by TrxR1/proteasome inhibition

**Fig. 5:**
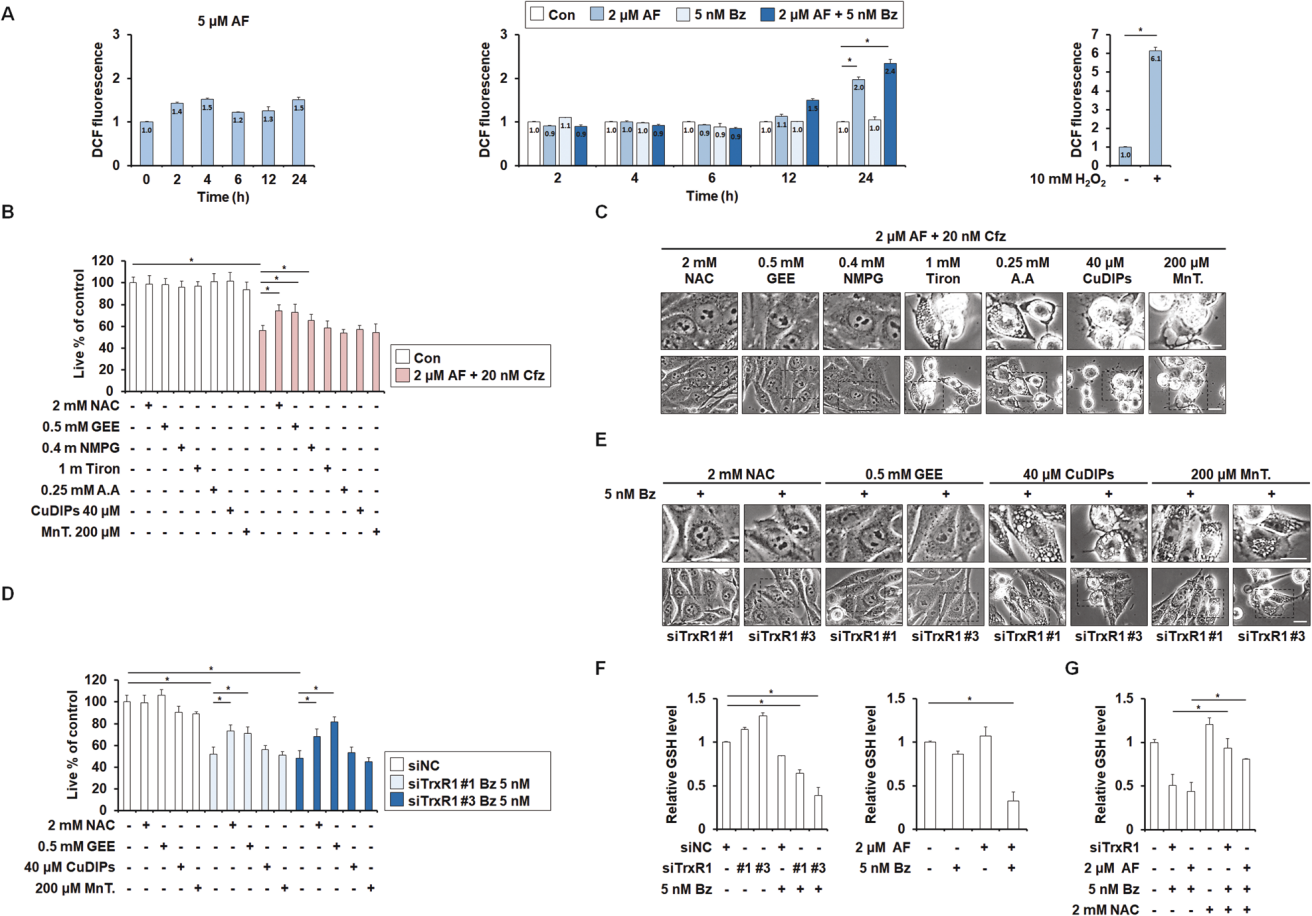
Disruption of thiol homeostasis rather than ROS generation is critical for the paraptosis induced by inhibition of TrxR1 and proteasome. **A** MDA-MB 435 S cells were treated with the indicated concentrations of AF and/or Bz for the indicated durations. Treated cells were incubated with CM-H_2_DCF-DA (DCF-DA) and subjected to flow cytometry. Cells treated with 10 mM H_2_O_2_ for 30 min were used as the positive control. Data are expressed as the fold change in DCF fluorescence intensity in treated cells compared to that in untreated cells. Data represent the means ± SD. (*n* = 3). One way-ANOVA and Bonferroni’s post hoc test. ^*^p < 0.005. **B, C** MDA-MB 435 S cells were pretreated with the indicated doses of N-acetylcysteine (NAC), glutathione reduced ethyl ester (GEE), N-(2-mercapto-propionyl)-glycine (NMPG), tiron, ascorbic acid (AA), Cu(II)(3,5-diisopropylsalicylate)2 (CuDIPs), or manganese (III) tetrakis (4-benzoic acid) porphyrin chloride (MnTBAP; MnT) and further treated with 20 nM Cfz plus 2 μM AF for 24 h. **D, E** MDA-MB 435 S cells transfected with siNC or siTrxR1 were treated with the indicated doses of NAC, GEE, CuDIPs, or MnT and further treated with 5 nM Bz for 24 h. **B**, **D** Cellular viability was assessed using IncuCyte, as described in the Materials and Methods. The percentage of live cells was normalized to that of untreated cells (100%). Data represent the means ± SD. (*n* = 9). One way-ANOVA and Bonferroni’s post hoc test. ^*^p < 0.05. **C, E** Cellular morphologies were observed by phase-contrast microscopy. Bars, 20 μm. **F** MDA-MB 435 S cells transfected with siNC or siTrxR1 were further treated with 5 nM Bz for 12 h (*left*) or treated with 5 nM Bz plus 2 μM AF for 12 h (*right*). **G** MDA-MB 435 S cells transfected with siNC or siTrxR1 were treated with 2 mM NAC and/or 5 nM Bz for 12 h, or treated with 2 mM NAC and/or AF plus Bz at the indicated concentrations for 12 h. **F**, **G** GSH levels were assessed, as described in the Materials and Methods.

Components of the Trx system, including TrxR1, contribute to rapid proliferation and pro-survival activity in cancer cells, particularly those facing increased oxidative stress [30]. Therefore, we first examined whether ROS generation is critical for the anticancer effect of TrxR1/proteasome inhibition. We performed flow cytometry using CM-H_2_DCF-DA, with H_2_O_2_ applied as a positive control for ROS generation. We found that treatment with 5 μM AF very weakly and transiently increased ROS levels at 2~4 h and then again at 24 h (Fig. 5A). In contrast, 2 μM AF increased ROS levels about 2.0 fold and 2 μM AF plus 5 nM Bz enhanced the increase to about 2.3 fold (both at 24 h) (Fig. 5A). These results suggest that the cell death induced by TrxR1/proteasome inhibition is accompanied by weak ROS generation. Since several antioxidants, including ascorbic acid and flavonoids, can directly bind and inactivate Bz [31], we assessed the effects of various antioxidants on the cell death induced by TrxR1/proteasome inhibition employing Cfz instead of Bz. Interestingly, we found that thiol-containing antioxidants, including N-acetylcysteine (NAC), glutathione reduced ethyl ester (GEE), and N-(2-mercapto-propionyl)-glycine (NMPG), markedly inhibited the cell death and vacuolation induced by 2 μM AF plus 20 nM Cfz, whereas this change was not seen with non-thiol ROS scavengers, such as tiron, ascorbic acid (AA), Cu(II)(3,5-diisopropylsalicylate)2 (CuDIPS), and manganese (III) tetrakis (4-benzoic acid) porphyrin chloride (MnTBAP; a superoxide dismutase mimetic) (Fig. 5B, C). In addition, NAC and GEE, very effectively blocked the cell death and vacuolation induced by TrxR1 knockdown/Bz, but CuDIPs, and MnTBAP, did not (Fig. 5D, E). Collectively, these results suggest that ROS generation may not critically contribute to this cell death. Since alteration of GSH homeostasis was reportedly correlated with increased AF sensitivity [32], we examined whether inhibition of TrxR1 and/or proteasome altered GSH levels. GSH levels were slightly increased by TrxR1 knockdown or 2 μM AF, slightly decreased by 5 nM Bz alone, and further reduced by TrxR1 knockdown/5 nM Bz or 2 μM AF/5 nM Bz (Fig. 5F). Pretreatment with NAC effectively recovered the GSH levels in cells treated with TrxR1 knockdown/Bz or AF/Bz (Fig. 5G). These results suggest that the death-blocking effect of thiol antioxidants, including NAC, may reflect the replenishment of intracellular GSH.

### ATF4 upregulation critically contributes to the paraptosis induced by TrxR1/proteasome inhibition

**Fig. 6:**
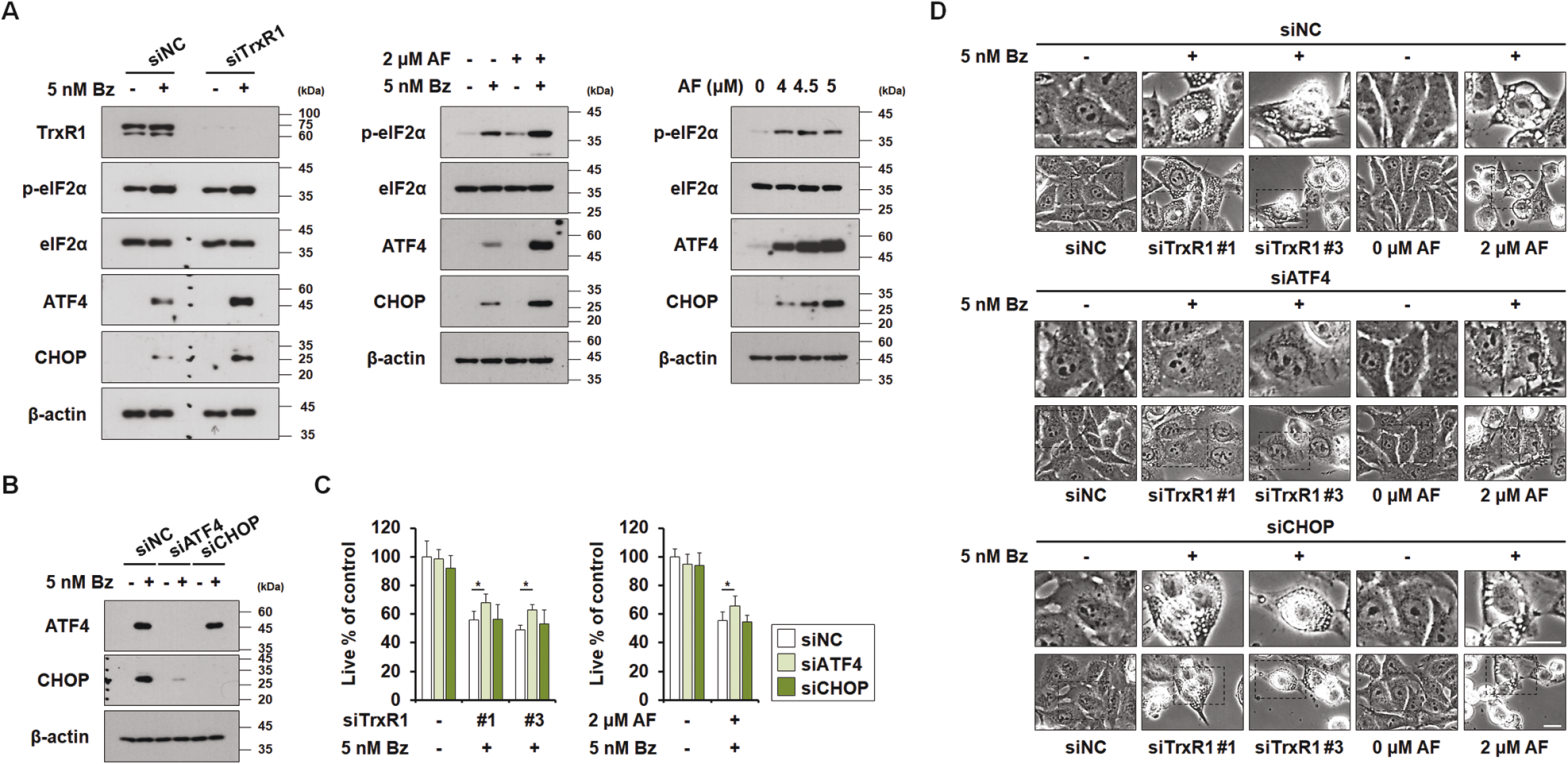
AF-mediated TrxR1 inhibition enhances Bz-induced ER stress, and ATF4 plays a critical role in the paraptosis induced by TrxR1/proteasome inhibition. **A** MDA-MB 435 S cells transfected with siNC or siTrxR1 were treated with 5 nM Bz for 12 h, or with the indicated concentrations of Bz and/or AF for 12 h. **B**, **D** MDA-MB 435 S cells transfected with siNC, ATF4-targeting siRNA (siATF4), or CHOP-targeting siRNA (siCHOP) were transfected with TrxR1 and further treated with 5 nM Bz for 24 h, or treated with the indicated concentrations of Bz and/or AF for 24 h. **A**, **B** Western blotting of the indicated proteins was performed using β-actin as a loading control. **C** Cellular viability was assessed using IncuCyte, as described in the Materials and Methods. The percentage of live cells was normalized to that of untreated cells (100%). Data represent the means ± SD. (*n* = 9). One way-ANOVA and Bonferroni’s post hoc test. ^*^p < 0.05. **D** Cellular morphologies were observed by phase-contrast microscopy. Bars, 20 μm.

The central mechanism of PI-mediated cell death involves the accumulation of toxic poly-ubiquitinated proteins and misfolded protein aggregates (i.e., proteotoxic stress [33, 34]), and PIs activate the integrated stress response (ISR) [35]. Therefore, we examined whether TrxR1 inhibition affects Bz-mediated ISR. We found that treatment with 5 nM Bz slightly increased the expression levels of ISR components, including phosphorylated eIF2α (p-eIF2α), ATF4, and CHOP (Fig. 6A). TrxR1 knockdown or 2 μM AF enhanced the Bz-mediated upregulation of p-eIF2α, ATF4, and CHOP in MDA-MB 435 S cells (Fig. 6A), indicating that TrxR1 inhibition enhances Bz-mediated ISR. AF treatment at paraptosis-inducing doses also markedly induced ISR, similar to the effect of TrxR1/proteasome inhibition. ATF4, the core effector of ISR [20, 36], is associated with diverse proteotoxic stress response pathways, including the mitochondrial unfolded protein response (UPR_mito_) [37, 38] and the ER-unfolded protein response (UPR_ER_) [39, 40]. CHOP is implicated in the paraptosis induced by dimethoxycurcumin [41] and indirubin-3’-monoxime [42]. When we examined the significance of ATF4 or CHOP, we found that knockdown of ATF4, but not CHOP, remarkably inhibited Bz-induced CHOP upregulation (Fig. 6B) and partially but significantly attenuated the vacuolation-associated cell death induced by TrxR1 knockdown/Bz or AF/Bz (Fig. 6C, D). These results suggest that ATF4 upregulation contributes to at least a portion of the paraptosis induced by TrxR1/proteasome inhibition.

### Upregulation of the ATF4/CHAC1 axis is critical for the paraptosis induced by TrxR1/proteasome inhibition through degrading glutathione

**Fig. 7:**
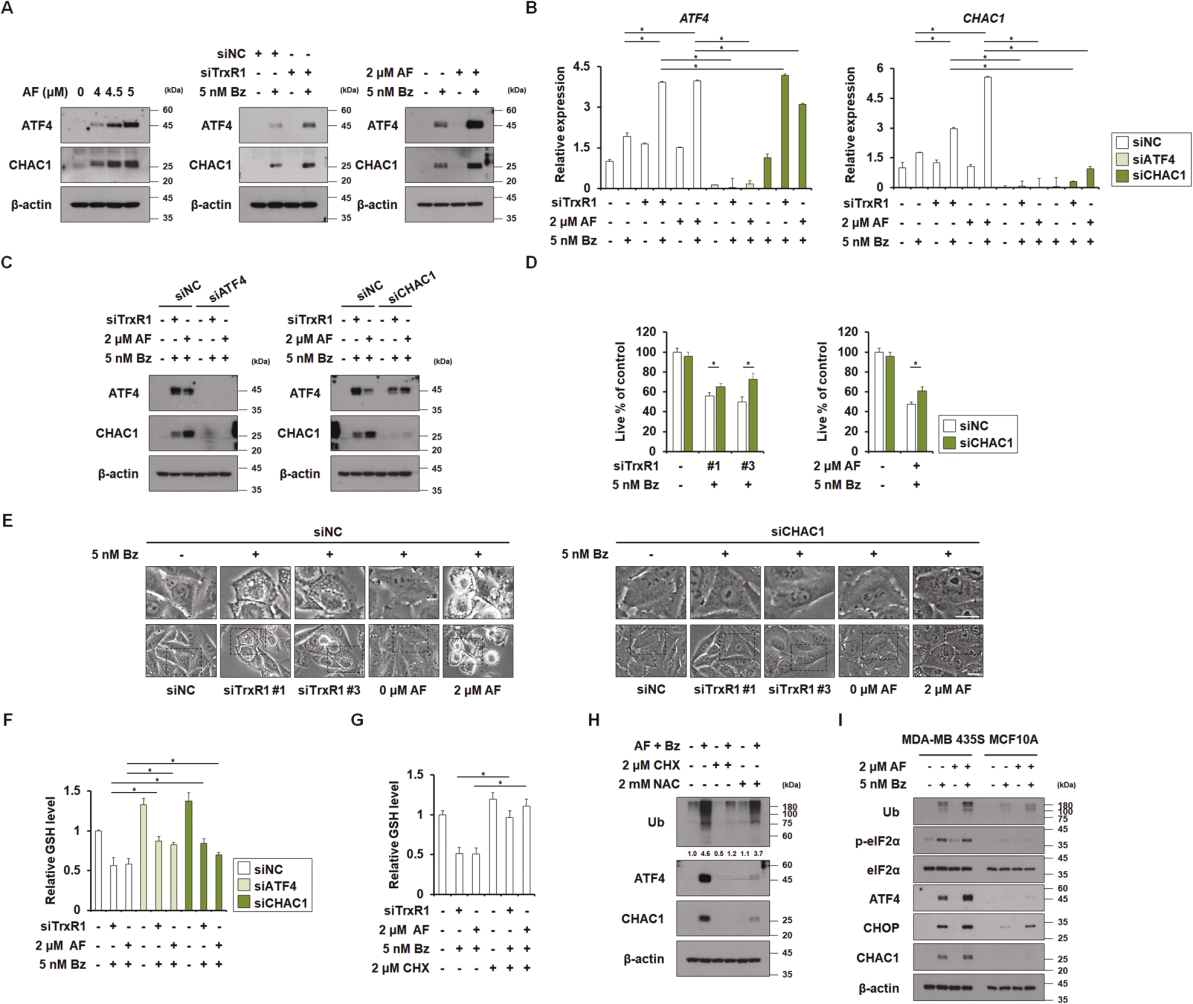
ATF4/CHAC1 critically contributes to the paraptosis induced by TrxR1/proteasome inhibition by degrading GSH. **A** MDA-MB 435 S cells were treated with the indicated concentrations of AF alone (*left*) or Bz and/or AF (*right*) for 12 h. MDA-MB 435 S cells transfected with siNC or siTrxR1 were treated with 5 nM Bz for 12 h (*middle*). MDA-MB 435 S cells transfected with siNC, siATF4, or CHAC1-targeting siRNA (siCHAC1) were cotransfected with TrxR1 and further treated with 5 nM Bz for 24 h (**B**–**E**) or 12 h (**F**), or treated with the indicated concentrations of Bz and/or AF for 24 h (**B**–**E**) or 12 h (**F**). **G**, **H** MDA-MB 435 S cells pretreated with 2 μM CHX or 2 mM NAC were treated with 2 μM AF plus 5 nM Bz for 12 h. **I** Cells were treated with 5 nM Bz and/or 2 μM AF for 12 h. **A**, **C**, **H**, **I** Western blotting of the indicated proteins was performed using β-actin as a loading control. **B** The mRNA levels of ATF4 or CHAC1 were assessed by qRT-PCR. **D** Cellular viability was assessed using IncuCyte, as described in the Materials and Methods. The percentage of live cells was normalized to that of untreated cells (100%). Data represent the means ± SD. (*n* = *9*). One way-ANOVA and Bonferroni’s post hoc test. ^*^p < 0.05. **E** Cellular morphologies were observed by phase-contrast microscopy. Bars, 20 μm. **F**, **G** GSH levels were assessed, as described in Materials and Methods.

Next, we investigated whether ATF4 critically contributes to the paraptosis induced by TrxR1/proteasome inhibition through modulation of its transcriptional target(s). As shown in Fig. 5, down-regulation of GSH was found to be critical for TrxR1/proteasome inhibition-induced paraptosis. Since CHAC1 (glutathione-specific gamma-glutamylcyclotransferase 1), a transcriptional target of ATF4, was shown to degrade GSH by cleaving it to 5-oxo-L-proline and a Cys-Gly dipeptide [43–45], we investigated the possible involvement of CHAC1 in this cell death. We found that AF dose-dependently increased the protein levels of CHAC1, which paralleled the expression of ATF4 (Fig. 7A). Either TrxR1 knockdown or 2 μM AF further enhanced the Bz-induced upregulation of CHAC1, in parallel with ATF4 upregulation, at the protein and mRNA levels (Fig. 7A, B). ATF4 knockdown effectively inhibited the CHAC1 upregulation induced by TrxR1 knockdown/Bz or AF/Bz at the mRNA and protein levels, whereas CHAC1 knockdown did not affect ATF4 expression (Fig. 7B, C). These results suggest that ATF4 acts upstream of CHAC1. Moreover, CHAC1 knockdown partially but significantly inhibited the cell death and vacuolation caused by TrxR1 knockdown/Bz or AF/Bz (Fig. 7D, E). Knockdown of ATF4 or CHAC1 significantly recovered the GSH levels reduced by TrxR1 knockdown/Bz or AF/Bz (Fig. 7F). These results suggest that the ATF4/CHAC1 axis contributes to at least a portion of the paraptosis induced by TrxR1/proteasome inhibition through GSH degradation. We also found that CHX pretreatment effectively restored the GSH levels (Fig. 7G). NAC pretreatment effectively inhibited the upregulation of poly-ubiquitinated proteins, ATF4, and CHAC1 induced by TrxR1 knockdown/Bz or AF/Bz, whereas CHX pretreatment almost completely inhibited these effects (Fig. 7H). Our results suggest that ATF4/CHAC1-mediated GSH degradation may aggravate proteotoxic stress via a vicious cycle. Therefore, dual TrxR1/proteasome inhibition triggers paraptosis by unresolved proteotoxic stress mediated through thiol imbalance. Interestingly, low-dose AF-induced enhancement of Bz-mediated ISR and CHAC1 upregulation observed in MDA-MB 435 S cells was not seen in MCF10A cells, suggesting that TrxR1/proteasome inhibition preferentially kills cancer cells via cancer-selective aggravation of proteotoxic stress (Fig. 7I).

**Fig. 8:**
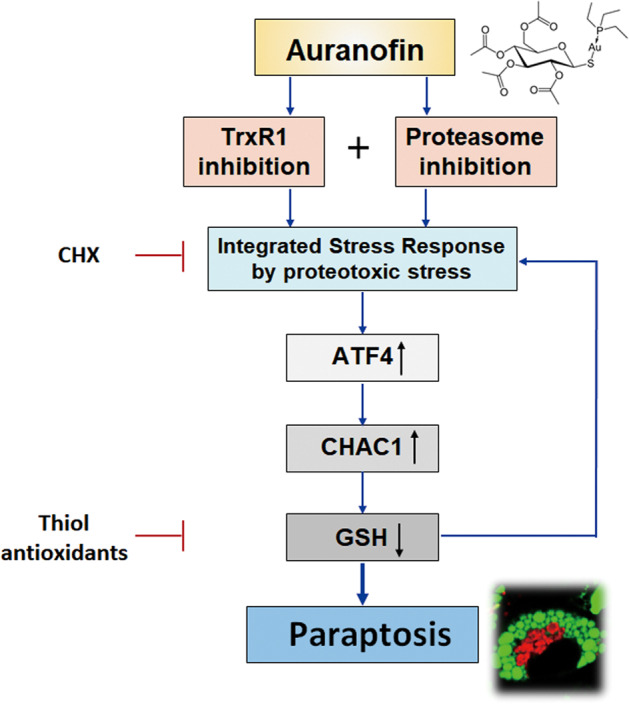
Hypothetical model for the mechanism underlying the paraptosis induced by auranofin or simultaneous inhibition of TrxR1 and proteasome. For the induction of auranofin-induced paraptosis, both TrxR1 inhibition and proteasome inhibition are required. Simultaneous TrxR1/proteasome inhibition triggers the ISR due to proteotoxic stress. In this process, GSH degradation, which is mediated by the ATF4/CHAC1 axis, critically contributes to inducing paraptosis by aggravating proteotoxic stress.

In summary, our results reveal that inhibition of both TrxR1 and proteasome is required for AF-induced paraptosis. Compared to high-dose AF, co-treatment with AF and PI at sub-lethal doses may be safer and yield a cancer-selective therapeutic effect. Mechanistically, simultaneous TrxR1/proteasome inhibition triggers ISR via proteotoxic stress that is mediated by ATF4/CHAC1 axis-mediated GSH degradation (Fig. 8).

## Discussion

Breast cancer is women’s most commonly diagnosed cancer [46]. Although advances in early detection and therapy have significantly improved breast cancer survival rates, most patients develop resistance to the interventions [47]. The major limitations of currently available chemotherapeutics are cancer’s drug resistance and toxic side effects [48–50]. Therefore, there is an unmet clinical need to develop innovative anticancer drugs with improved clinical efficacy and tolerability. In this respect, drug repurposing is a promising and effective strategy [51, 52], and AF, an FDA-approved antirheumatic drug [1], is being investigated for potential therapeutic application in cancer [2]. We show here for the first time that AF induces paraptosis in breast cancer cells via simultaneous inhibition of TrxR1 and proteasome. Many reports indicate that AF has a complex and multifaceted mode of action. For example, AF has been shown to induce apoptosis in various types of cancer cells [53–56], and to induce apoptosis and necrosis in Jurkat [57], HeLa [58], and lung cancer cells [59]. There are indications that mitochondrial oxidative stress is a central event in cells undergoing AF-induced apoptosis and necrosis [60]. Whereas 2-3 μM AF induces apoptosis in Jurkat cells, higher doses cause cell death through necrosis [57]. Furthermore, while AF mainly induces apoptosis in p53 R175H-expressing non-small cell lung cancer (NSCLC), it induces ferroptosis in p53 R273H-expressing NSCLC cells [61]. These results suggest that various factors, including the doses of AF, cancer cell type, and genetic background of cells, may affect the determination of the final cell death mode induced by AF [62]. Further study is needed to clarify the exact mechanism(s) through which AF induces multiple cell death modes in cancer. Our study measured cell viability by fixing, washing, propidium iodide staining, and IncuCyte imaging of AF-treated cells. Propidium iodide-stained attached cells were considered alive since dead cells were removed during the washing and fixing. We found that treatment with 1~5 µM AF dose- and time-dependently reduced cell viability, whereas 20 µM AF rapidly and dramatically reduced viability (Supplementary Fig. 2A). Next, we graphed the % of dead cells, which was obtained by subtracting the % of live cells (graphed in Supplementary Fig. 2A) from 100 (Supplementary Fig. 2B). Propidium iodide uptake in cells is known to occur via the loss of plasma membrane integrity in necrotic cells. We further performed the image-based cell death assay of propidium iodide-stained adherent mammalian cells following the protocol proposed by Szali and Engedal [63]. MDA-MB 435 S cells were treated with various concentrations of AF together with 5 µg/ml propidium iodide and observed over time. Few PI-positive cells were seen in groups treated with 5 µM AF for 24 h, whereas almost all cells treated with 20 µM AF for 24 h were PI-positive (Supplementary Fig. 2C). We quantitatively assessed cell death by calculating the ratio of red area to total cell area using the IncuCyte ZOOM software following the protocol proposed by the previous study [63]. We found that 1~5 µM AF induced far less cell death than 20 µM AF (Supplementary Fig. 2D). In addition, the extent of propidium iodide positivity induced by 4~5 µM AF (Supplementary Fig. 2D) was much lower than that shown in Fig. 1B, which was assessed by calculating the percentage of dead cells using the percentage of attached (live) cells (Supplementary Fig. 2A). Therefore, we speculate that AF may induce necrosis at 20 µM AF in MDA-MB 435 S cells. Further characterization of this cell death mode will be needed going forward. In contrast, our results suggest that cells treated with 4~5 µM AF tend to maintain their plasma membrane integrity until death-induced cellular detachment, suggesting that paraptosis differs from necrosis in terms of plasma membrane integrity.

TrxRs are selenoproteins with a highly nucleophilic selenocysteine residue at the active site, making them prone to irreversible inhibition by AF even at nanomolar concentrations [3–5]. In addition to TrxR, proteasome-associated DUBs were implicated in the anticancer effect of AF [7]. Proteasomal inhibition by AF was reported to require 2-3 fold higher doses than that required for TrxR1 inhibition^5^. Here, we show that TrxR1 inhibition by TrxR1 knockdown or 2 µM AF or proteasome inhibition by 5 nM Bz or 20 nM Cfz did not individually kill MDA-MB 435 S cells. However, 2 µM AF plus PI or TrxR1 knockdown plus PI effectively induced paraptosis, similar to the effect of 5 µM AF. These results suggest that AF-induced paraptosis requires the dual inhibition of TrxR1 and proteasome. However, 4~5 µM AF was cytotoxic to the tested breast cancer and non-malignant MCF10A cells. AF reacts with exposed nucleophilic cysteine or selenocysteine residues [64]. Therefore, high-dose AF may inhibit multiple targets essential for the survival of normal cells, including IKK-β [65, 66], STAT3 [67, 68], and protein kinase C iota [69, 70], in addition to TrxR and proteasome. We speculated that a combined regimen could minimize toxicity while optimizing dosing. Indeed, we herein show that, in contrast to high-dose AF, sub-lethal doses of AF and PI were selectively cytotoxic to cancer cells, sparing normal cells.

AF inhibits cytosolic and mitochondrial TrxR (TrxR1 and TrxR2), two selenoenzymes for the Trx pathway [3]. Previously, concentrations above 5 μM AF were shown to effectively inhibit both TrxR1 and TrxR2, whereas AF at low concentrations (ranging from 0.1 to 1 μM) was more specific for TrxR1 compared to TrxR2 [3]. Mitochondrial TrxR2 was shown to be targeted by AF, leading to mitochondrial oxidative stress and apoptosis [71]. Therefore, it will be interesting to investigate whether TrxR2 inhibition sensitizes Bz-mediated cell death by inducing paraptosis in a manner similar to the effect of TrxR1 inhibition, or by inducing ROS-mediated apoptosis or necrosis.

It is intriguing to speculate on why co-targeting of TrxR1 and proteasome induces cancer-selective paraptosis. Rapidly growing cancer cells exhibit elevated ROS levels and accelerated protein synthesis [72, 73]. TrxR1, a critical modulator of protein redox homeostasis, has emerged as a potential target for cancer therapy, especially in tumor types susceptible to oxidative stress [74]. The proteasome-mediated degradation pathway is an important target for cancer therapy, since proteasome activity is essential for tumor cell proliferation and drug resistance [75, 76]. Therefore, the preferential cytotoxicity of dual TrxR1/proteasome inhibition may reflect the higher dependency of cancer cells on TrxR1 and proteasome to maintain proteostasis. On the other hand, the inability of TrxR1 or proteasome mono-inhibition to kill breast cancer cells may indicate the presence of a cellular backup system and/or resistance mechanisms. In our study, TrxR1 knockdown or 2 µM AF alone slightly increased GSH levels without inducing cell death. A previous study showed that GSH could reduce Trx1 when TrxR1 activity was lost [77], suggesting that GSH may serve as a backup for TrxR1. In addition, tumors lacking TrxR1 were highly susceptible to pharmacological GSH deprivation in vitro and in vivo [78]. Although PIs are currently used to treat multiple myeloma (MM), most MM patients demonstrate drug-resistant relapse following long-term Bz treatment. Furthermore, the anticancer efficacy of PIs in solid tumors has not been satisfactory [79]. Elevated TrxR1 levels correlate with the acquisition of Bz resistance in MM, and TrxR1 inhibition using AF or TrxR1 silencing reverses Bz resistance [80]. Additionally, increasing GSH levels was shown to abolish Bz-induced cytotoxicity in MM cells [81]. Thus, GSH is causatively linked to the resistance to both TrxR1 and proteasome inhibition. We found that 2 µM AF/Bz or TrxR1 knockdown/Bz induces cell death in breast cancer cells via GSH depletion and upregulation of ISR components. In this process, the ATF4/CHAC1 axis critically contributes to cell-death-related GSH degradation. The NAC-mediated recovery of GSH levels effectively blocked TrxR1/proteasome inhibition-induced ISR and paraptosis. These results suggest that there is a vicious cycle between proteotoxic stress and GSH degradation in the cell death induced by TrxR1/proteasome inhibition. Cytosolic TrxR1 was reported to be critical in reducing non-native disulfides in the ER [82]. Therefore, TrxR1 inhibition could contribute to the misfolding of proteins entering the secretory pathway and the accumulation of misfolded proteins within the ER. GSH also plays a crucial role in native disulfide bond formation within the ER [83], and GSH-dependent proofreading occurs during mitochondrial disulfide-mediated oxidative protein folding [84]. Proteostatic disruption, including impairment of protein thiol homeostasis [11, 14–17] and proteasomal inhibition [18–21], has been critically implicated in paraptosis. The vacuolization observed during paraptosis is believed to reflect the influx of water into the ER and mitochondria when osmotic pressure is increased by misfolded proteins accumulated within these organelles [11]. Therefore, TrxR1/proteasome inhibition may trigger proteotoxicity-mediated paraptosis by concomitantly targeting both protein thiol homeostasis and proteasome.

In summary, a combined regimen of reduced doses of AF and Bz may address the potential issues of side effects triggered by high-dose AF and resistance of solid tumors to PI. In addition, the ability of Bz to synergistically enhance the anticancer effects of AF provides a rationale to reposition the latter FDA-approved drug for cancer therapy.

## Materials and methods

### Chemicals and antibodies

Chemicals and reagents were obtained as follows: bortezomib (Bz) and carfilzomib (Cfz) from Selleckchem (Houston, TX, USA); Necrostatin-1, bafilomycin A1, N-acetyl-L-cysteine (NAC), and H_2_O_2_ from Sigma-Aldrich (St Louis, MO, USA); cycloheximide (CHX), SP600125, PD98059, and Manganese (III) tetrakis (4-benzoic acid) porphyrin chloride (MnTBAP) from Calbiochem (EDM Millipore Corp., Billerica, MA, USA); Auranofin (AF) from AdipoGen (Liestal, CH, Switzerland); MitoTracker-Red (MTR), propidium iodide, and 5-(and-6)-chloromethyl-2′,7′-dichlorofluorescein diacetate (CM-H_2_DCF-DA) from Molecular Probes (Eugene, OR, USA); z-VAD-fmk from R&D systems (Minneapolis, MN, USA); Glutathione reduced ethyl ester (GSH-OEt) from Chemodex (Gallen, CH, Switzerland); SB203580 from Tokyo Chemical Industry (TCI, Tokyo, Japan); CDDO-ME from Cayman (Michigan, USA). The following primary antibodies were used: TrxR1 (HPA001395) from Sigma-Aldrich; Ubiquitin (sc-8017) and β-actin (sc-47778) from Santa Cruz Biotechnology (Santa Cruz, CA, USA); ATF4 (#11815), p-eIF2α (#9721), eIF2α (#9722), and CHOP/GADD153 (#2895) from Cell Signaling Technology (Danvers, MA, USA); CHAC1 (15207-1-AP) from Proteintech (Rosemont, IL, USA). The secondary antibodies (rabbit IgG HRP(G-21234) and mouse IgG HRP (G-21040)) were from Molecular Probes.

### Cell culture

MDA-MB 435 S, MDA-MB 231, BT549, and MCF-10A cells were directly purchased from American Type Culture Collection (ATCC, Manassas, VA, USA) and tested for mycoplasma contamination. MDA-MB 435 S cells were cultured in DMEM, and MDA-MB 231 and BT549 cells were cultured in RPMI-1640 medium supplemented with 10% fetal bovine serum (FBS) and 1% antibiotics (GIBCO-BRL, Grand Island, NY, USA). MCF-10A cells were maintained in DMEM/F12 supplemented with pituitary extract, insulin, human epidermal growth factor, hydrocortisone, and cholera toxin (Calbiochem). Cells were incubated in 5% CO_2_ at 37 °C.

### Cell viability assay

Cells were cultured in 24-well or 48-well plates and treated as indicated. The cells were then fixed with methanol/acetone (1:1) at −20 °C for 5 min with PBS, and stained with propidium iodide (PI; final concentration, 1 μg/mL) at room temperature for 10 min. The plates were imaged on an IncuCyte device (Essen Bioscience, Ann Arbor, MI, USA) and analyzed using the IncuCyte ZOOM 2016B software. The processing definition of the IncuCyte program was set to recognize attached (live) cells by their red-stained nuclei. The percentage of live cells was normalized to that found in untreated control cultures (100%).

### TrxR activity assay

TrxR activity was determined using a thioredoxin reductase colorimetric assay kit (Cayman Chemical). MDA-MB 435 S cells (1.5 × 10^5^/ml) in 60 mm culture dishes were treated with the indicated concentration of AF or Bz for 20 min and then washed with PBS. Cells were harvested with cold 50 mM potassium phosphate buffer (pH 7.4) containing 1 mM EDTA and then sonicated. Homogenized cells were centrifuged at 10,000 x *g* for 15 min at 4 °C and the supernatant was added to the wells of a 96-well plate, along with NADPH and 5,5′-dithiobis(2-nitrobenzoic acid) (DTNB). TrxR activity was monitored as the ability to use NADPH to reduce 5,5’-dithio-bis(2-dinitrobenzoic acid) to 5-thio-2-nitrobenzoic acid (TNB); this produced a yellow product that was measured at 405 nm. Measurement of TrxR activity by DTNB reduction in the presence and absence of a TrxR inhibitor enabled us to correct non-thioredoxin reductase-independent DTNB reduction.

### Isobologram analysis

To investigate how the combinations of PIs and AF affected the cancer cell lines, dose-dependent effects were determined for each compound alone and with a fixed concentration of the other co-treated agent. The interactions of the PIs and AF were quantified by determining the combination index (CI), by the following classic isobologram equation: CI = (D)1/(Dx)1 + (D)2/(Dx)2, where (Dx)1 and (Dx)2 indicate the individual doses of PIs and AF, respectively, required to produce an effect, and (D)1 and (D)2 are the doses of PIs and AF, respectively, that produce the same effect when applied in combination. From this analysis, the combined effects of the two drugs can be summarized as follows: CI < 1 indicates synergism; CI = 1 indicates summation (additive and zero interaction); CI > 1 indicates antagonism.

### Morphological examination of the ER and mitochondria

The stable cell lines expressing the fluorescence specifically in the ER lumen (YFP-ER cells) were previously described [18]. After treatments, YFP-ER cells were stained with 100 nM MitoTracker-Red (MTR) for 10 min, and morphological changes of the ER and mitochondria were observed under a K1-Fluo confocal laser scanning microscope (Nanoscope Systems, Daejeon, Korea).

### Immunoblot analyses

Cells were washed in PBS and lysed in sodium dodecyl sulfate (SDS) sample buffer (125 mM/L Tris [pH6.8], 1% SDS, 20% glycerol, and 2% β-mercaptoethanol). The extracts were boiled for 5 min, separated by SDS-PAGE, and transfected to a Polyvinylidene fluoride membrane (Millipore). After blocking nonspecific binding sites for 30 min with 5% skim milk, membranes were incubated for 1 h with antibodies. Membranes were washed three times with TNET buffer (50 mM Tris-HCl [pH7.4], 150 mM NaCl, 5 mM EDTA, 0.05% Tween20) and incubated further for 1 h with horseradish peroxidase-conjugated anti-rabbit or mouse. Visualization of protein bands was accomplished using ECL (Advansta).

### Measurement of proteasome activity employing Ub^G76V^-GFP

Cells transfected with Ub^G76V^-GFP were cultured for 48 h in a 24-well plate and treated with the indicated agents for 12 h. The plates were imaged on an IncuCyte device, and the fluorescence intensities were analyzed using the IncuCyte ZOOM 2016B software.

### Small interfering RNA-mediated gene silencing

siRNA Negative Control (siNC) (Stealth RNAi^TM^, 12935300) was purchased from Invitrogen (Carsbad, CA, USA). *TrxR1 (TXNRD1)* targeted siRNAs were purchased from Origin (Rockville, MD, USA): siTrxR1 #1 (target sequence GAAUGGACGAUUCCGUCAAGAGAUA); siTrxR1 #2 (target sequence CAGCAGUGAUGAUCUUUUCUCCUUG); siTrxR1 #3 (target sequence ACAAGUACAUCUGCGAUCAACUCTA) (SR304982). *ATF4 (CREB-2)* targeted siRNA was from Santa Cruz: siATF4 (target sequence CCACUCCAGAUCAUUCCUU, GGAUAUCACUGAAGGAGAU, and GUGAGAAACUGGAUAAGAA, sc-35112). *CHOP (DDIT3)* targeted siRNA was synthesized from Invitrogen: siCHOP (target sequence GAGCUCUGAUUGACCGAAUGGUGAA). *CHAC1* targeted siRNA was synthesized from Genolution (Seoul, Korea): siCHAC1 (target sequence GAUCAUGAGGGCUGCACUU). The pairs of siRNA oligonucleotides were annealed and transfected to cells using the RNAiMAX reagent (Invitrogen), according to the manufacturer’s instructions. To confirm successful siRNA-mediated knockdown, Western blotting of the proteins of interest was performed.

### Measurement of reactive oxygen species (ROS) generation

Treated cells were incubated with 10 μM of CM-H_2_DCF-DA (DCF) for 30 min at 37 °C, trypsinized, washed, resuspended in PBS, and subjected to flow cytometry using a CytoFLEX (Beckman Coulter Life Sciences, Brea, CA, USA). Mean DCF fluorescence values are measured and expressed as the fold change with regard to the background level of untreated cells.

### Measurement of intracellular GSH levels

Intracellular GSH levels were analyzed using a Glutathione Assay kit (Cayman, Michigan, USA). MDA-MB 435 S cells plated to 6-well plates (20 × 10^4^ cells/well) were treated with AF and/or Bz for 12 h, washed with PBS, trypsinized, and centrifuged at 2000 x *g* for 10 min at 4 °C. Cells were lysed by sonication in 1X MES buffer (0.2 M 2-(N-morpholino)methanesulfonic acid, 0.5 mM K_2_HPO_4_, 1 mM EDTA (pH 6.0)) and centrifuged. Samples were then mixed with an equal volume of 0.1 µg/ml metaphosphoric acid and centrifuged to precipitate protein; and 200 mM triethanolamine was added to supernatants. Samples were then transferred to a 96-well plate (50 µL/well) and the reaction was started by adding assay buffer with GSH reaction mixture (containing NADP + , glucose-6-phosphate, glucose-6-phosphate reductase, glutathione reductase, and 5,5’-dithiol-bis-2-nitrobenzoic acid). Production of 5-thio-2- nitrobenzoic acid was measured for 25 min using a microplate reader (Synergy2, BioTek, Winooski, VT, USA). A portion of lysate, taken from before protein precipitation, was set to determine protein concentration.

### RNA isolation and quantitative real-time RT-PCR (qRT-PCR)

Total RNA was isolated from MDA-MB 435 S cells transfected with siNC, siTrxR1, siATF4, or siCHAC1 by using TRIzol® reagent (10296010, Thermo Scientific (Waltham, MA, USA)), according to the manufacturer’s instructions. To perform quantitative real-time RT-PCR (qRT-PCR), cDNA was synthesized from 1 μg of total RNA of each sample using an M-MLV cDNA Synthesis kit (EZ006S, Enzynomics (Daejeon, Korea)). qRT-PCR was performed using a Bio-Rad Real-Time PCR System (Bio-Rad, Richmond, CA, USA). Relative expression levels were determined with the ΔΔCt method [85] and normalized to the mean of the housekeeping gene GAPDH. All primer sequences of qRT-PCR were listed in Supplementary Table.

### Statistical analysis

All data are presented as mean ± SD (standard deviation) from at least three separate experiments. To perform statistical analysis, GraphPad Prism (GraphPad Software Inc, Sandiego, CA) was used. Normality of data was assessed by Kolmogorov–Smirnov tests and equal variance using Bartlett’s test. For a normal distribution, statistical differences were determined using an analysis of variance (ANOVA) followed by Bonferroni multiple comparison test. If the data were not normally distributed, Kruskal–Wallis test was performed followed by Dunn’s test. ^*^p < 0.05 was considered statistically significant.

## Supplementary information

Check list

Revised Supplementary Information

Supplementary table

## Data Availability

All data and information concerning this study will be made available upon request.
