# Supplementary material for: Dual inhibition of thioredoxin reductase and proteasome is required for auranofin-induced paraptosis in breast cancer cells
Source: Cell Death Dis. 2023 Jan 19;14(1):42. doi: 10.1038/s41419-023-05586-6 (PMC9852458; doi:10.1038/s41419-023-05586-6)
Supplement: Supplementary file 2 — Revised Supplementary Information [file 41419_2023_5586_MOESM2_ESM.docx]

**Supplementary Figures**

**
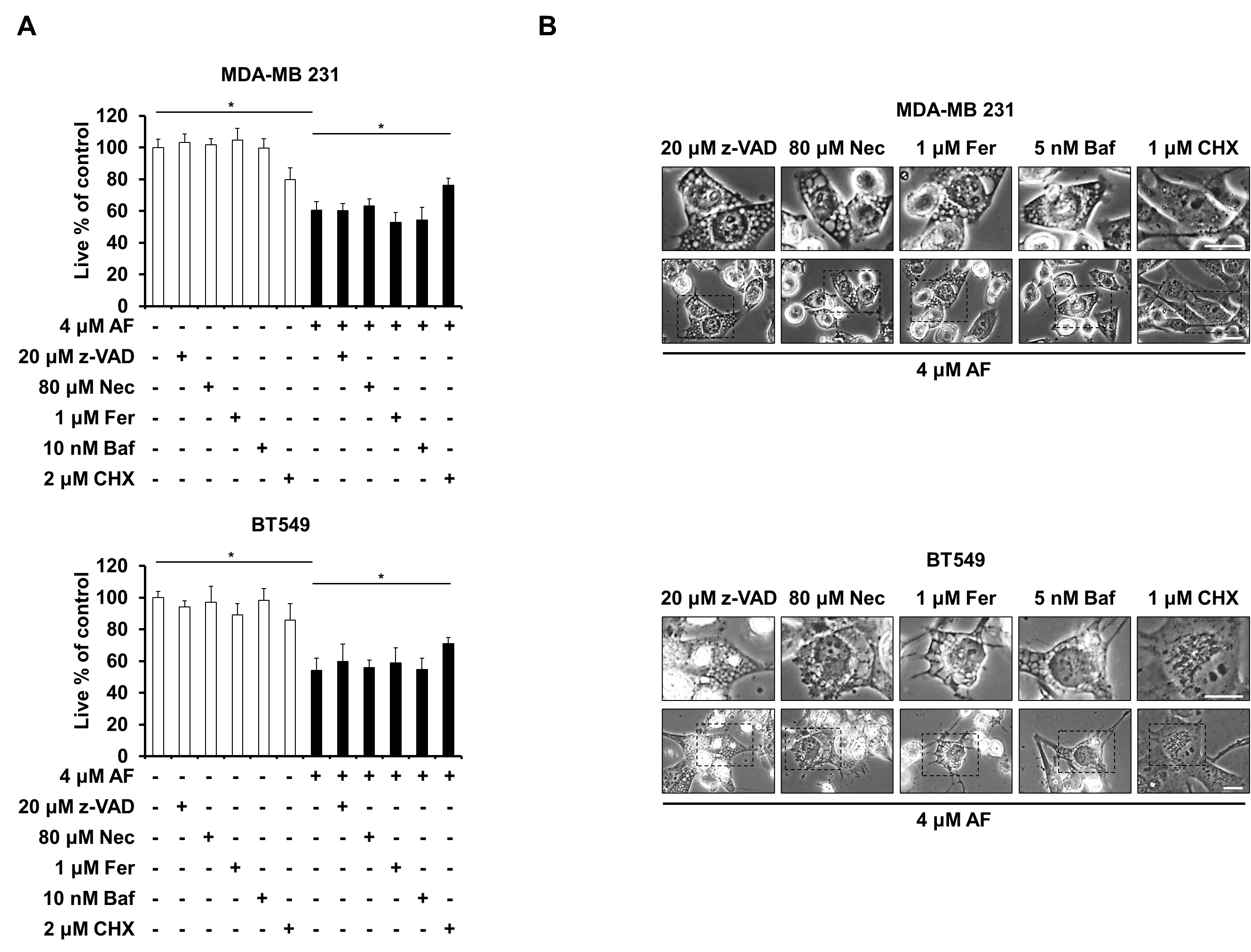
**

**Supplementary Fig. 1 Only CHX but not inhibitors of other death modes blocks AF-induced vacuolation-associated cell death in MDA-MB 231 and BT549 cells.**

**A, B** Cells pretreated with the inhibitors of various death modes at the indicated concentrations were further treated with 4 μM AF for 24 h. **A** Cellular viability was assessed using IncuCyte, as described in the Materials and Methods. The percentage of live cells was normalized to that of untreated cells (100%). Data represent the means ± SD. (n = 9). One way-ANOVA and Bonferroni’s post hoc test. * p < 0.05. **B** Cellular morphologies were observed by phase-contrast microscopy. Bars, 20 μm.

**
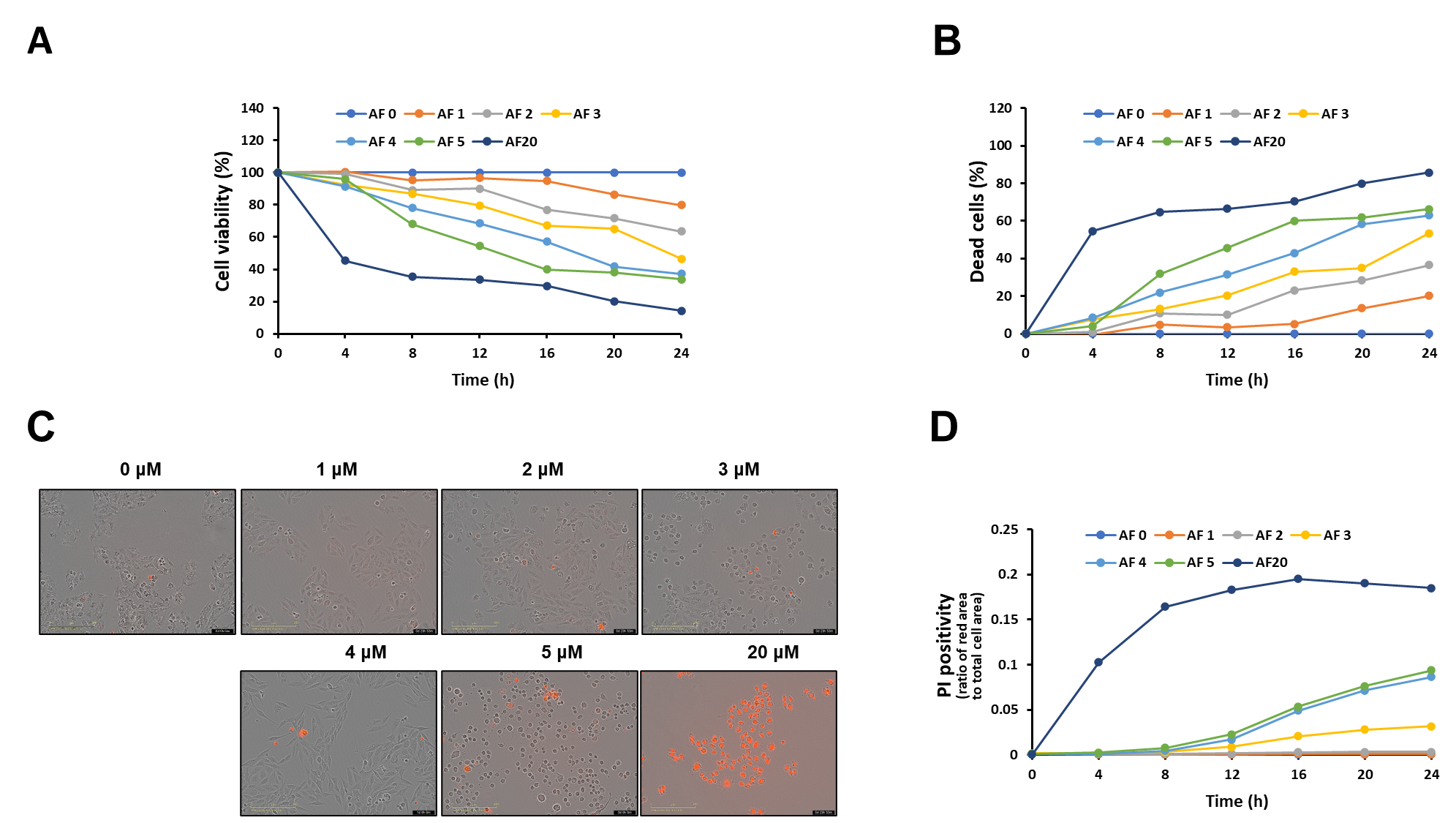
**

**Supplementary Fig. 2. Comparison of the results obtained from two death assays using PI and IncuCyte.**

**A** MDA-MB 435S cells treated with different doses of AF for various periods were fixed with methanol/acetone, washed, stained with 1 µg/ml propidium iodide, imaged on an IncuCyte Zoom instrument, and analyzed using the IncuCyte software. The processing definition of the IncuCyte program was set to recognize attached (live) cells by their red-stained nuclei. The percentage of live cells was normalized to that found in untreated control cultures (100%). **B** The percentage of dead cells was plotted by subtracting the percentage of live cells shown in Figure **1A** from 100%**. C, D** MDA-MB 435S cells were incubated with different doses of AF for 24 h (**C**) or stained with 2.5 µg/ml PI for various periods (**D**). **C** Cellular images obtained with an IncuCyte device. Bar, 20 µm. **D** The ratio of red fluorescent (propidium iodide-positive) to total cell confluence plotted over time was analyzed using the IncuCyte Zoom system and denoted in the graph. Cell confluence was taken as the portion of the surface covered by cells, as determined by phase-contrast images.


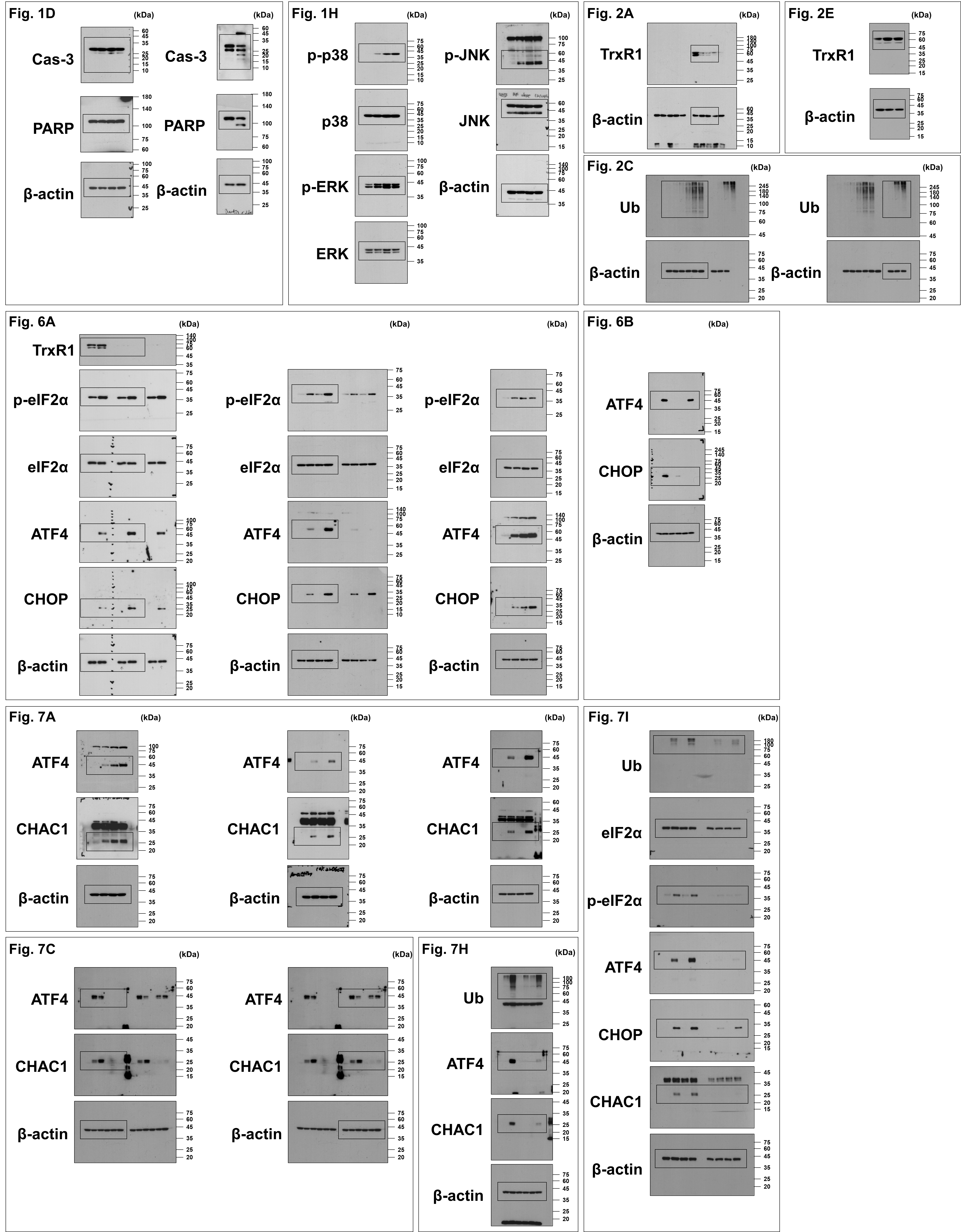


**Supplementary Fig. 3 The full scan for this study’s western blot images.**

The black-lined boxes on the western data indicate the cropped images used in Fig. 1D, 1H, 2A, 2C, 2E, 6A, 6B, 7A, 7C, 7H, and 7I.
