# Supplementary material for: Dual inhibition of thioredoxin reductase and proteasome is required for auranofin-induced paraptosis in breast cancer cells
Source: Cell Death Dis. 2023 Jan 19;14(1):42. doi: 10.1038/s41419-023-05586-6 (PMC9852458; doi:10.1038/s41419-023-05586-6)
Supplement: Supplementary file 3 — Supplementary table [file 41419_2023_5586_MOESM3_ESM.docx]

**Table S1. The list of PCR primer sequences**

| Gene | Primer sequences | |
| --- | --- | --- |
|  | Forward (5’-3’) | Reverse (5’-3’) |
| TXNRD1 | ACACAAAGCTTCAGCATGTCA | CAATTCCGAGAGCGTTCC |
| ATF4 | CTCCGGGACAGATTGGATGTT | GGCTGCTTATTAGTCTCCTGGAC |
| CHAC1 | GTGGTGACGCTCCTTGAAGA | TTCAGGGCCTTGCTTACCTG |
| GAPDH | GAGTCAACGGATTTGGTCGT | TGGAAGATGGTGATGGGATT |
